# *Bacillus siamensis* 3BS12-4 Extracellular Compounds as a Potential Biological Control Agent against *Aspergillus flavus*

**DOI:** 10.4014/jmb.2402.02053

**Published:** 2024-06-30

**Authors:** Patapee Aphaiso, Polson Mahakhan, Jutaporn Sawaengkaew

**Affiliations:** 1 Graduate School, Khon Kaen University, Khon Kaen 40002, Thailand; 2 Department of Microbiology, Faculty of Science, Khon Kaen University, Khon Kaen 40002, Thailand

**Keywords:** *Aspergillus flavus*, aflatoxin B_1_, *Bacillus siamensis*, extracellular compounds, hydrolytic enzyme, peanut seeds

## Abstract

*Aspergillus flavus*, the primary mold that causes food spoilage, poses significant health and economic problems worldwide. Eliminating *A. flavus* growth is essential to ensure the safety of agricultural products, and extracellular compounds (ECCs) produced by *Bacillus* spp. have been demonstrated to inhibit the growth of this pathogen. In this study, we aimed to identify microorganisms efficient at inhibiting *A. flavus* growth and degrading aflatoxin B_1_. We isolated microorganisms from soil samples using a culture medium containing coumarin (CM medium) as the sole carbon source. Of the 498 isolates grown on CM medium, only 132 bacterial strains were capable of inhibiting *A. flavus* growth. Isolate 3BS12-4, identified as *Bacillus siamensis*, exhibited the highest antifungal activity with an inhibition ratio of 43.10%, and was therefore selected for further studies. The inhibition of *A. flavus* by isolate 3BS12-4 was predominantly attributed to ECCs, with a minimum inhibitory concentration and minimum fungicidal concentration of 0.512 g/ml. SEM analysis revealed that the ECCs disrupted the mycelium of *A. flavus*. The hydrolytic enzyme activity of the ECCs was assessed by protease, β-1,3-glucanase, and chitinase activity. Our results demonstrate a remarkable 96.11% aflatoxin B_1_ degradation mediated by ECCs produced by isolate 3BS12-4. Furthermore, treatment with these compounds resulted in a significant 97.93% inhibition of *A. flavus* growth on peanut seeds. These findings collectively present *B. siamensis* 3BS12-4 as a promising tool for developing environmentally friendly products to manage aflatoxin-producing fungi and contribute to the enhancement of agricultural product safety and food security.

## Introduction

*Aspergillus flavus* is one of the major spoilage molds commonly contaminating agricultural products such as corn, rice, and peanuts [1]. It is a serious global health and economic problem, leading to direct and indirect disadvantages. Direct disadvantages include depressed commodity prices and agricultural commodities that do not meet specified standards. Indirect disadvantages include the adoption of *A. flavus* contamination data as a trade barrier [2]. Furthermore, toxicogenic *A. flavus* strains produce mycotoxins, the most common of which are aflatoxins, secondary metabolites that are a group of derivatives of difuranocournarins/difurocoumarins and peptone or lactone rings [3].

Aflatoxins are the most potent naturally occurring hepatocarcinogens and mutagens in humans and animals, and can lead to DNA mutations, immunosuppression, and hepatotoxicity [4]. Among the four types of aflatoxins (B_1_, B_2_, G_1_, and G_2_), aflatoxin B_1_ is the most toxic and potent carcinogen. The International Agency for Research on Cancer (IARC) has classified aflatoxin B_1_ as a Group 1 carcinogen to humans [5]. The Food and Agriculture Organization (FAO) has estimated that approximately 25% of agricultural products produced worldwide are contaminated with mycotoxins [6]. Various strategies have been suggested to be responsible for the inhibitory effects of microorganisms on *A. flavus* growth and aflatoxin B_1_ contamination, including chemical, physical, and biological methods, or combinations thereof.

In recent years, research on the sustainable control of *A. flavus* contamination has focused on eliminating or preventing the various factors that promote *A. flavus* growth. Three main methods, using chemical, physical, and biological approaches, have been employed to achieve this goal. The various chemical and physical methods that have been applied and evaluated for controlling *A. flavus* and aflatoxin B_1_ include alkalization, ammonization, heat, and gamma radiation. However, these methods are not always effective or feasible for farmers to implement [7]. In addition, the chemical and physical methods of controlling *A. flavus* and aflatoxin B_1_ contamination have several disadvantages. They lack wide acceptance because they can reduce the quality of agricultural products, and even produce byproducts that can spontaneously form aflatoxins with detrimental effects on human and animal health [8]. The biological methods of controlling *A. flavus* and aflatoxin B_1_ contamination are a promising approach for both pre-harvest and post-harvest control. These methods use plants, herbs, insects, essential oils, and microorganisms. Microbial extracts have advantages over plant extracts because microbes can grow rapidly, produce large amounts of bioactive compounds in a short time, and are environmentally friendly [9, 10]. Microorganisms have been shown to produce massive quantities of bioactive compounds or antimicrobial agents that can eliminate and detoxify *A. flavus* and aflatoxin B_1_. Several microbial strains have been identified for this purpose, including *Bacillus* spp. [11], *Lactobacillus* spp. [12], *Stenotrophomonas maltophilia* [13], *Trametes versicolor* [14], and a few yeasts, such as *Saccharomyces cerevisiae* [15]. Of these microorganisms, *Bacillus* spp. are particularly well-known for producing bioactive compounds that can successfully eliminate *A. flavus* growth and aflatoxin B_1_ contamination.

*Bacillus* spp. is gram-positive, rod-shaped, endospore-forming bacteria that are commonly found in soil, air, and water. Their endospore-forming ability enables them to be stable and tolerant during feed manufacturing, agricultural storage, and stress conditions. This is an important advantage over other microbial species, as endospores can withstand environmental stress conditions and regain viability under suitable conditions. *Bacillus* spp. are versatile microorganisms with applications in almost every sector. For example, Chaucheyras-Durand and Durand (2010) [16] demonstrated that *Bacillus* spp. can be successfully applied in other types of animal farming, such as aquaculture and domestic livestock. Several reports have shown that *Bacillus* spp. can produce a large number of bioactive compounds for the control of fungal diseases, such as lipopeptides (three families of lipopeptides: surfactin, iturin, and fengycin) [17], bacteriocins and bacteriocin-like inhibitory substances [18], and cell wall-degrading enzymes [19].

Here, we focused on isolating *Bacillus* spp. capable of producing bioactive compounds with antifungal activity against *A. flavus*. Bioactive compounds from the chosen isolate (3BS12-4) were first characterized, and then an investigation of their growth-inhibiting mechanisms against *A. flavus* using scanning electron microscopy was performed. Additionally, we evaluated the isolatés degradation capabilities, *i.e.*, its ability to remove aflatoxin B_1_ and its biocontrol potential on peanut seeds, including inhibition of *A. flavus* growth and aflatoxin B1 degradation.

## Materials and Methods

### Isolation of Potential Extracellular Compound-Producing Bacteria

Eighty-two soil samples were collected from different areas in Thailand, including Wang Saphung district, Loei Province; Romklao Kalapapruek Garden, Khon Kean University; and Ban Kong subdistrict, Nong Ruea district, Khon Kean Province. GPS coordinates, temperature, and soil characteristics regarding the samples were also recorded. Ten-gram samples of each soil were suspended in 90 ml of sterile 0.9% NaCl and boiled at 90°C for 10 min [20]. The soil samples were then cross-streaked in nutrient agar (NA) petri dishes and grown at 37°C for 24 h. Colonies that appeared on NA were isolated as single cells and subsequently transferred to fresh NA slants for further experiment.

### Screening of the Isolated Bacteria for Growth Inhibition of *A. flavus*

Screening for bacteria capable of inhibiting *A. flavus* growth was carried out using a minimal salt medium containing 0.1% coumarin (CM medium), which was prepared as described by Hassan *et al*. (2016) [21]. The pH of the CM medium was adjusted to 7, as reported by Guan *et al*. (2008) [13]. Twenty milliliters of CM medium were prepared per petri dish. The isolates from the experiment described above were point inoculated onto CM plates using a sterile toothpick with a diameter of 2.00 mm. The plates were incubated at 37°C until visible colonies appeared, or for up to 5 days. Subsequently, only bacteria that grew on CM medium were selected. The individual colonies were grown in fresh CM medium for the antagonistic assay.

### Antifungal Activity by Extracellular Compound-Producing Bacteria

The antifungal activity of the isolated bacteria against *A. flavus* was evaluated using a dual culture assay on potato dextrose agar medium (PDA). The dual culture assay was performed as described by Xia *et al*. (2017) [22], with modifications. Mycelial discs 6 mm in diameter were cut from the growing edge of a 3-day-old *A. flavus* culture using a sterile cork-borer and placed in the center of fresh PDA petri dishes (containing 20 mL of medium per petri dish). The petri dishes were incubated at 30°C for 24 h. Single colonies of each isolate, and sterile water used as a control, were point inoculated on PDA petri dishes at two equidistant sites 3 cm from the center. The petri dishes were incubated at 30°C for 3 days. The diameters of the fungal colonies were measured to calculate the inhibition ratios using the following formula:

$$\text{Inhibition ratio (\%) = }\frac{\text{(r-r')×100}}{\text{r}}\text{,}$$

where r (mm) is the growth of the fungus from the center of the colony towards the edge of the petri dish, and r' (mm) is the growth of the fungus from the center of the colony towards the center of the bacteria tested.

### Identification of Bacteria Isolates

The isolate 3BS12-4 was identified using morphological tests and 16S rRNA gene sequence analysis. Morphological tests, including Gram staining, colony color, and colony shape, were performed on these bacterial isolates. To identify the bacteria, the 16S rRNA gene sequence of the isolate of interest was amplified by PCR using the universal primers 27F (5'-AGA GTT TGA TCM TGG CTC AG-3') and 1492R (5'-TAC GGY TAC CTT GTT ACG ACT T-3'). The nucleotide sequences were subjected to the (BioEdit Sequence Alignment Editor 7.2). Complete sequences were analyzed by using 16S-based ID services to compare with type strains in the EZBioCloud database. 16S rRNA gene sequences were aligned with the ClusstalW, and a phylogenetic tree was performed using the neighbor-joining method including 1000 bootstrap replicates.

### Production of Potential Extracellular Compounds for Growth Inhibition of *A. flavus*

The production of extracellular compounds was performed according to Rao *et al*. (2017) [23] with slight modification. The bacterial isolate was activated in 50 ml of nutrient broth (NB) at 37°C for 16 h. Then, 5 ml (10%v/v) of the preculture was inoculated into 50 ml of NB and incubated at 37°C for 48 h. After incubation, the culture supernatant and bacterial precipitate were harvested by centrifugation at 7,000 ×*g* for 10 min at 4°C. The culture supernatant was considered the extracellular compound, and the bacterial precipitate was considered the intracellular compound. The bacterial precipitate was washed twice with 0.05 M sodium phosphate buffer, pH 7.0. After resuspension, the bacterial precipitate was disintegrated using ultrasonication to obtain intracellular extracts. The extracellular and intracellular compounds were concentrated by freeze-drying, and then resuspended in 0.05 M sodium phosphate buffer at pH 7.0 for use in determining the minimum inhibitory concentration (MIC) and minimum fungicidal concentration (MFC).

### Determination of Minimal Inhibitory and Fungicidal Concentrations

The minimal inhibitory concentration (MIC) and minimal fungicidal concentration (MFC) of various extracellular and intracellular compounds from isolate 3BS12-4 were measured using a 96-well microtiter plate assay. Flat-bottom, polystyrene 96-well plates (Eppendorf, Germany) were used. To measure the MIC, 100 μl of various concentrations of crude extracellular compound (final concentrations of 1.024, 0.512, 0.256, 0.128, 0.064, 0.032, 0.016, 0.008, and 0.004 g/ml dissolved in 0.05 M sodium phosphate buffer, pH 7.0) were mixed with *A. flavus* (100 μl, 1 × 10^6^ spores/ml) [24]. The crude extracellular compound of each experimental type was distributed in 9 wells of a 96-well microtiter plate. The plates were incubated at 30°C for 72 h. The MFC of the fungus was measured following the method of Ghannoum *et al*. (2011) [25]. The total inhibition of crude extracellular compound exhibited by each clear well from the MIC measurement was poured onto a PDA petri dish to determine the colony count. The absence of *A. flavus* growth on PDA plates, particularly at low concentrations, indicated that these PDA plates represented the MFC values.

### Scanning Electron Microscopy (SEM) Evaluation

The morphological alterations of *A. flavus* growth samples from the previous MIC and MFC tests caused by extracellular compounds were investigated and confirmed using a field emission scanning electron microscope (Quattro S FEG E-SEM; Thermo Fisher Scientific, USA). A total of 1 × 10^6^ spores/ml *A. flavus* was co-inoculated with 0.512 g/ml of extracellular compounds. The samples preparation was fixed, washed, dehydrated, critical point dried, and observed. Untreated extracellular compounds served as control. The morphology of *A. flavus* in treated and untreated samples was compared to assess the extracellular compounds for *A. flavus* inhibition. The detailed SEM protocol was described by Li *et al*. (2022a) [26].

### Hydrolytic Enzyme Analysis (Protease, β-1,3-Glucanase, and Chitinase Activity)

The 0.512 g/ml crude extracellular compound of the bacterial isolate was dissolved in 0.05 M sodium phosphate buffer, pH 7.0, to assay protease, chitinase, and β-1,3-glucanase activity. These enzymatic activities were determined using modified versions of the Folin reagent method [27] for protease activity assay and the Dinitro salicylate (DNS) method [28] for chitinase and β-1,3-glucanase activity assays. Total protein was determined by Lowry method [29].

### Determination of Protease Activity in Crude Extracellular Compound

Protease activity was measured using a modified method with casein as the substrate. The reaction mixture contained 1 mL of diluted crude enzyme and 1 ml of 2% casein in 0.05 M sodium phosphate buffer, pH 7.0. The mixture was incubated at 45°C for 15 min and the reaction was stopped by the addition of 2 ml of 10%trichloroacetic acid (TCA). The mixture was left at 4°C for 1 h to allow for complete or almost complete protein precipitation. The reaction mixture was centrifuged at 7,000 ×*g* for 10 min, and the supernatant was measured for tyrosine concentration using the Lowry method [29]. One unit of protease was defined as the amount of protease that released 1 μg/ml of tyrosine from casein per minute under experimental conditions.

### Determination of β-1,3-Glucanase Activity in Crude Extracellular Compound

The β-1,3-glucanase activity was assessed by incubating a 250 μl reaction mixture containing 1.0% (w/v) laminarin in 0.05 M sodium phosphate buffer, pH 7.0, as the substrate, and 250 μl of crude enzyme at 45°C for 3 h. The reaction was stopped by adding 0.5 ml of DNS reagent and boiling for 20 min, followed by immediate cooling. The absorbance at 540 nm was measured. One unit of β-1,3-glucanase was defined as the amount of β-1,3-glucanase that released 1 μmol of glucose per minute under experimental conditions.

### Determination of Chitinase Activity in Crude Extracellular Compound

Chitinase activity was assessed by preparing a reaction mixture composed of 250 μl of 1% (w/v) colloidal chitin dissolved in 0.05 M sodium phosphate buffer, pH 7.0, as the substrate, and 250 μl of crude enzyme. The reaction mixture was incubated at 45°C for 3 h and stopped by adding 0.5 ml of DNS. The reaction mixture was then boiled for 15 min and immediately cooled. Distilled water (2.5 ml) was added, and the mixture was centrifuged at 7,000 ×*g* for 10 min. The absorbance of the supernatant was measured at 520 nm. One unit of chitinase was defined as the amount of chitinase that released 1 μmol of N-acetylglucosamine (GlcNAc) under the experimental conditions.

### Aflatoxin Removal by Extracellular Compounds

The removal of extracellular compounds (ECCs) from isolate 3BS12-4 to aflatoxin B_1_ was performed according to Rao *et al*. (2017) [23] with slight modification. Briefly, a 1 ppm stock solution of standard aflatoxin B_1_ (Sigma Aldrich, USA) was diluted with methanol. The experiments were carried out using 1.5 ml Eppendorf tubes containing 25 μl of aflatoxin B_1_ and 475 μl of ECCs (final concentration of aflatoxin B_1_ with 50 ppb and final concentration of ECCs with 0.512 g/ml). The reaction mixture was inoculated at 30°C for 3 days. The control treatment consisted of a reaction mixture tube without the ECCs, which was substituted for 0.05 M sodium phosphate buffer, pH 7.0. After incubation, the reaction mixture was assessed for residual aflatoxin B_1_ and analyzed by Agilent 1100 series, USA.

### Aflatoxin B_1_ Analysis by HPLC

For quantification of residual aflatoxin B_1_, HPLC/FLD (high-performance liquid chromatography/fluorescence detector) was performed according to AOAC Official Method 991.31 [30]. The aflatoxin B_1_ sample was extracted from residual aflatoxin B_1_ dissolved in methanol for analysis using an immunoaffinity column, then evaporated until dry under nitrogen gas. Afterwards, the residual aflatoxin B_1_ was derivatized using a mixture of trifluoroacetic acid with glacial acetic acid and filtered through a sterile nylon syringe filter (pore size 0.2 μm). The extracted aflatoxin B_1_ samples were analyzed with an Agilent 1100 Series HPLC equipped with a C18 column, 4.6 × 150 mm, 5 μm pore and fluorescence detector (FLD). The HPLC condition followed a flow rate of 1.0 ml/min with a run time of 15 min, column temperature of 40°C, and injection volume of 20 μl. The analysis was performed using a mobile phase of water: acetonitrile: methanol (72: 14: 14, v/v/v). The excitation and emission wavelengths were detected at 365 and 440 nm, respectively. The calibration curve of aflatoxin B_1_ standard (0.5, 1.5, 10, 20, and 30 ppb) at various concentrations was used to quantify the aflatoxin B_1_ content. The limit of detection (LOD) and the limit of quantitation of aflatoxin B_1_ were 0.5 and 1.5 ppb, respectively. The percentage of residual aflatoxin B_1_ was measured using the following formula: (C - T)/C × 100%, where C and T (ppb) are control treatment and treatment concentration, respectively.

### Biocontrol of Extracellular Compound on Peanut Seeds Under Storage Condition

Biocontrol on peanut seeds for inhibiting *A. flavus* growth and aflatoxin B_1_ degradation were performed according to Einloft *et al*. (2021) [31] with slight modification. Twenty grams of peanut seeds were sterilized using an autoclave and poured on a plate. The peanut seeds were then mixed with 1 ml of *A. flavus* (1 × 10^6^ spore/g peanut seeds) and 1 ml of extracellular compound (0.512 μl/g peanut seeds) and incubated at 30°C for 10 days. The plates were agitated by hand. For the control, 1 ml of 0.05 M sodium phosphate buffer, pH 7.0, was added on the plate instead of extracellular compound. After incubation, the samples were evaluated for fungal viable plate counts, and diluted samples in 0.01% peptone were spread on a petri dish containing 20 ml of Dichloran Rose Bengal Chloramphinicol agar (DRBC).

## Results and Discussion

### Isolation and Screening of Bacterial Strain for *A. flavus* Growth Inhibition

**Fig. 1:**
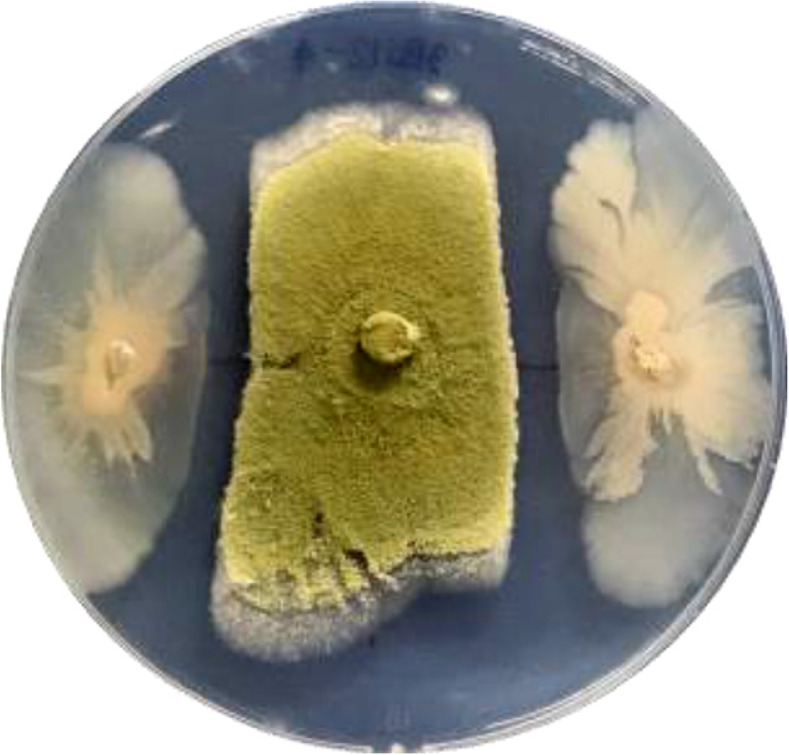
The antifungal activity of isolate 3BS12-4 with *A. flavus* growth at 30°C for 24 h.

In this study, 82 soil samples were collected from three different sources. Bacteria were isolated on nutrient agar (NA) medium, and 498 isolates were obtained. Of these isolates, 132 were able to grow on coumarin (CM medium) as a sole carbon source. Nineteen isolates were tested for antifungal activity against *A. flavus* by confronting incubation assay on potato dextrose agar (PDA) plates. Isolate 3BS12-4 exhibited the highest antifungal activity, with an inhibition ratio of 43.1%. Fig. 1 shows the antifungal activity of isolate 3BS12-4 against *A. flavus*. A 6.00-mm agar plug of *A. flavus* was placed at the center of a potato dextrose agar (PDA) plate, and isolate 3BS12-4 was inoculated at two equidistant sites 3 cm from the center. The plate was incubated at 30°C for 24 h. The image shows that the growth of *A. flavus* is inhibited around isolate 3BS12-4. The inhibition zone is approximately 1.5 cm wide. This indicates that isolate 3BS12-4 produces antifungal compounds that can inhibit the growth of *A. flavus*. Isolate 3BS12-4 was isolated from anthill soil in Wang Saphung district, Loei Province, Thailand. It is a rod-shaped, spore-forming, gram-positive bacterium. The partial 16S rRNA gene sequence of isolate 3BS12-4 was compared to the existing database in the EZBioCloud website. The sequences were aligned using ClustalW and we found that isolate 3BS12-4 showed 99.93% sequence similarity with *Bacillus siamensis* KCTC13613 (Accession No. AJVF01000043).

Environmental samples rich in microbial diversity, such as animal feces, soils, water, air, and cereal grains, were selected as sources of microorganisms that could efficiently inhibit *A. flavus* and degrade aflatoxin B_1_. To isolate and screen bacterial strains from these environmental samples, a medium containing coumarin (CM medium) as the sole carbon source was used as the primary screening method [13]. Aflatoxins are derivatives of coumarin and therefore, bacteria that can utilize CM as a carbon source may also be able to metabolize aflatoxins. In this study, *B. siamensis* 3BS12-4 exhibited antifungal activity against *A. flavus* with an inhibition ratio of 43.1%. Similarly, *B. subtilis* JSW-1 showed fungistatic activity against *A. flavus* with a percentage inhibition ratio of 58.3% [22]. The antifungal activity of *B. subtilis* CW14 against *A. ochraceus* 3.4412 was 33.3%, which was lower than that of *B. siamensis* 3BS12-4 [32]. Other studies have reported the biological control of *A. flavus* and aflatoxin B_1_ by various bacteria [26, 33, 34].

### Evaluation of Minimum Inhibitory Concentration (MIC) and Minimum Fungicidal Concentration (MFC)

**Fig. 2:**
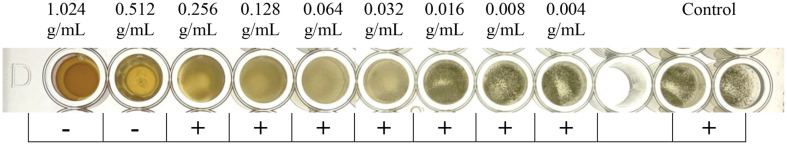
MIC of extracellular compounds from *B. siamensis* isolate 3BS12-4 against *A. flavus* determined using a 96-well microtiter plate assay. The figure legend defines well turbidity as an indicator of fungal growth, with “ + ” signifying growth and “ - ” indicating no growth.

In this study, we evaluated the antifungal activity of extracellular compounds produced by *B. siamensis* strain 3BS12-4 against *A. flavus* using a 96-well microtiter plate assay. The minimum inhibitory concentration (MIC) and minimum fungicidal concentration (MFC) were determined. Intracellular compounds exhibited no antifungal activity. The MIC values of the extracellular compounds ranged from 0.004 to 1.024 g/ml, with a significant inhibitory effect observed at a concentration as low as 0.512 g/ml. Serial dilution of the extracellular compounds resulted in a decrease in turbidity, with the initial observation at 0.256 g/ml (Fig. 2). The MFC values of the extracellular compounds were also determined to be 0.512 g/ml on petri dishes. At this concentration, there was a complete absence of fungal growth. Serial dilution demonstrated no growth at 0.256 g/ml and below. Detailed results of these experiments are presented in Table 1.

Zhang *et al*. (2008) [35] reported similar results, that a cell-free culture filtrate of *B. subtilis* B-FS06 inhibited the growth and spore germination of *A. flavus*. Chen *et al*. (2019) [34] observed that the three synthesized peptides from the supernatant of *B. megaterium* showed relatively high inhibition of *A. flavus* at a concentration of 32 mg/well. Similar observations were made by Li *et al*. (2022b) [36], who extracted the supernatant of *B. velezensis* E2 for utilization as an antifungal substance (lipopeptides). Their results showed that the minimum concentration of lipopeptides necessary to have an inhibitory effect was 0.5 mg/ml, and 2.5 mg/ml. While the extracellular compounds of *B. siamensis* 3BS12-4 demonstrate potential antifungal activity against *A. flavus* growth, complete elimination of *A. flavus* using only these compounds is likely to be challenging.

### SEM Analysis

**Fig. 3:**
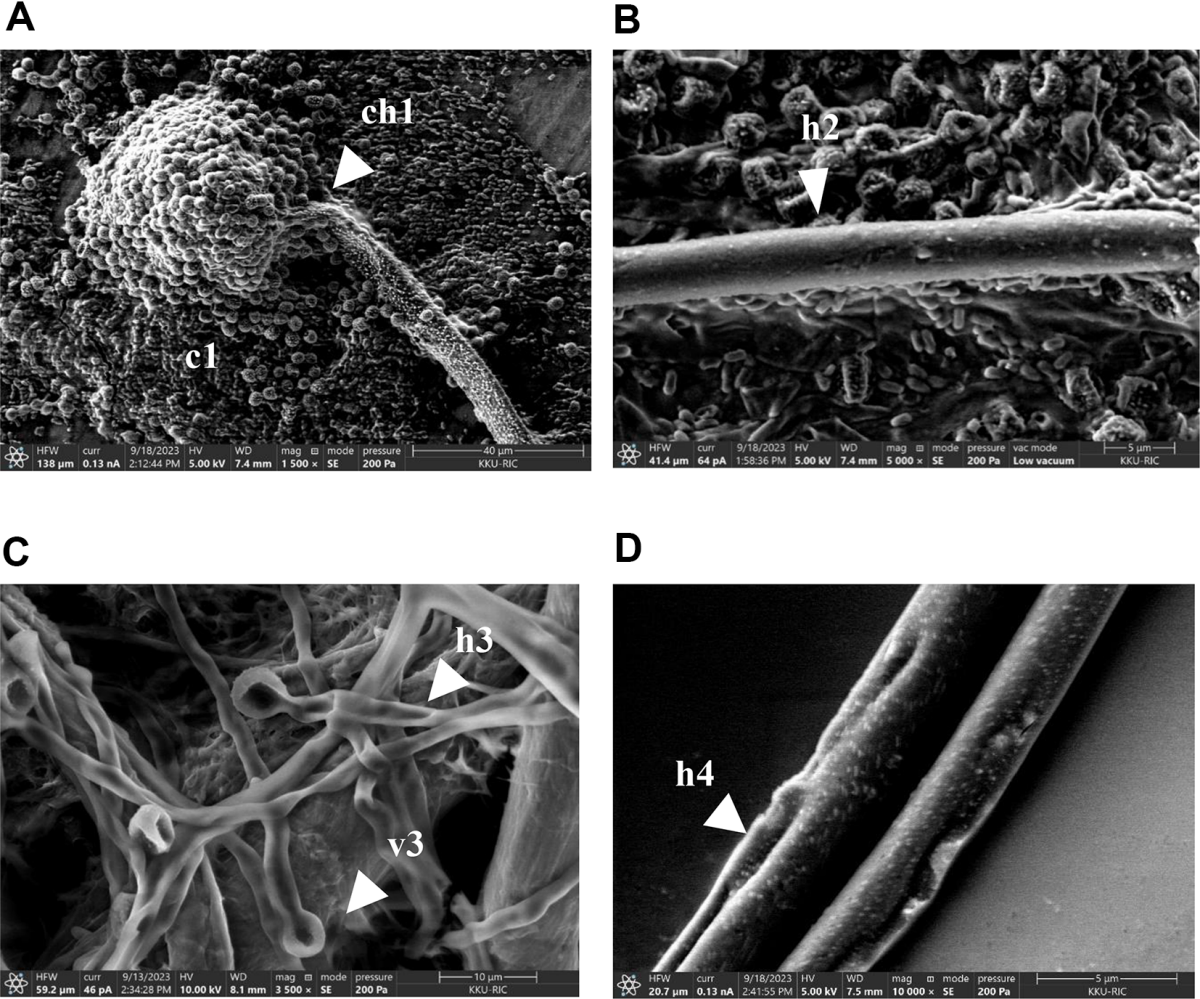
SEM of *A. flavus* showing morphological of hypha and conidiospores after co-inoculation with extracellular compound of *B. siamensis* 3BS12-4 at 30°C for 72 h. (**A, B**) control (without extracellular compound), (**C, D**) *A. flavus* treated with extracellular compound of *B. siamensis* 3BS12-4 at 0.512 g/ml.

The morphological alterations of *A. flavus* induced by extracellular compounds from *B. siamensis* 3BS12-4 were investigated using scanning electron microscopy (SEM) after incubation at 30°C for 72 h, as shown in Fig. 3. This analysis aimed to assess the biological control potential of these compounds. SEM revealed significant morphological alterations in *A. flavus* treated with extracellular compounds from *B. siamensis* 3BS12-4 (Fig. 3C and 3D), compared to the untreated control (Fig. 3A and 3B). Treated hyphae exhibited deformations (h3), folding (h3), denting (h4), and deformed vesicles (v3), suggesting cell wall damage. Notably, conidiospores appeared injured and lacked the ability to germinate. In contrast, the control group displayed healthy conidiophores (ch1), conidia (c1), and hyphae with a rough surface (h2), indicating normal morphology. These findings align with reports on other antifungal-producing bacteria, such as *Bacillus subtilis* fmbj, which synthesize antifungal substances like bacillomycin D. Similar to our observations, investigations utilizing SEM have demonstrated that bacillomycin D damages the mycelium and conidiospores of *A. flavus*, leading to leakage of cytoplasm and organelles, resulting in the formation of empty holes within the fungal cells [33]. Our results are consistent with those reported by Li *et al*. (2022b) [36], who observed morphological changes in *A. flavus* treated with lipopeptides from *B. velezensis* E2. Their study showed that *A. flavus* hyphae treated with lipopeptides lacked conidia, were twisted, and collapsed, and exhibited smoother and more swollen mycelium compared to the control. Akocak *et al*. (2015) [37] assessed the morphological change caused by cell wall-degrading enzymes, such as chitinase from *Pseudomonas fluorescens* PB_2_7, *B. cereus* B_1_, and *B. thuringiensis* K1. Their results showed an ability of chitinase to alter and deform the cell wall and mycelial morphology of *A. flavus* and partially exude organelles. The co-incubation mycelium dry weight of *A. flavus* with those bacteria was reduced by more than 70% (range 70-85%). In an earlier study, biocontrol of lactic acid bacteria (LAB) produced 1-pentanal, with volatile organic compounds demonstrating the highest antifungal activity with an MIC value of 40 μl/l and showing mycelial shriveling with a deformed conidial head in *A. flavus* [26]. Our experiment demonstrated that the extracellular compounds produced by *B. siamensis* 3BS12-4 have the potential to damage the cell wall of *A. flavus* mycelium and conidiospores. This damage likely leads to deformation and disruption of internal cytoplasm and organelles, ultimately causing leakage of these cellular components from the fungal cells.

### Hydrolytic Enzyme Assay

The production of hydrolytic enzymes, including protease, β-1,3-glucanase, and chitinase, was investigated from the extracellular compound of *B. siamensis* 3BS12-4. This study found the highest activities of protease, β-1,3-glucanase, and chitinase at 4.48 ± 0.09, 15.35 ± 0.02, and 1.21 ± 0.005 units/mg protein, respectively. Our findings align with those of Thakaew and Niamsup (2013) [38], who demonstrated that *B. subtilis* BCC 6327 produces hydrolytic enzymes (protease, β-1,3-glucanase, and chitinase) that damage and destroy the cell wall of *A. flavus*. Similarly, the *B. safensis* RGM 2450 and *B. siamensis* RGM 2529 strains possess genes encoding these bioactive compounds. These strains inhibit fungal growth through the secretion of diffusible compounds [39]. Peberdy (1990) [40] highlighted that chitin, β-1,3-glucan, and mannoprotein are major structural components of fungal cell walls. This study demonstrates that *B. siamensis* can produce hydrolytic enzymes that target these components, potentially deforming phytopathogenic fungi. Notably, *Lysinibacillus macrolides*, identified as the most antagonistic bacterial strain in this study, exhibited the highest chitinase productivity (26.45 U/L). This enzyme activity likely contributed to the observed hydrolysis and pitting of the *A. flavus* cell wall, ultimately leading to damage and inhibited conidia formation [41]. Schönbichler *et al*. (2020) [42] demonstrated the effectiveness of β-1,3-glucanases in degrading fungal cell walls by hydrolyzing β-1,3-glucosidic linkages in glucans, a major component alongside chitin. This, combined with the observed increase in protease activity during the growth of *B. subtilis* *natto* on fungal biomass, suggests a potential synergistic effect in breaking down the complex structure of the fungal cell wall [43]. Based on the combined results of SEM analysis and hydrolytic enzyme activity measurements, we posit that the observed antifungal effect of the extracellular compounds from *B. siamensis* 3BS12-4 is partially mediated by hydrolytic enzymes. While these enzymes demonstrate activity against *A. flavus*, their specific roles remain uncharacterized. Nonetheless, the observed deconstruction of *A. flavus* hyphae and conidia suggests that the extracellular hydrolytic enzymes contribute to the antifungal activity. Furthermore, the possibility of other proteins or enzymes contributing to the observed antifungal activity warrants further investigation.

### Aflatoxin Removal by Extracellular Compounds

This study used HPLC/FLD analysis to assess the ability of extracellular compounds from isolate 3BS12-4 to remove aflatoxin B_1_. Compared to the control, the presence of these compounds led to a progressive decrease in aflatoxin B_1_ concentration, from 32.93 ppb to 1.28 ppb for 3 days of incubation, representing a remarkable 96.11%removal achieved by the extracellular compounds. Cho *et al*. (2000) [44] reported a 95.5% reduction in aflatoxin B_1_ from 5 days when treated with *Azospirillum* sp., which was less than that of *B. siamensis* 3BS12-4 for aflatoxin B_1_ degradation. Consistent with previous findings, *B. subtilis* JSW-1 demonstrated the ability to degrade aflatoxin B_1_, achieving a 67.2% reduction after 3 days of incubation at 30°C [22]. Previously, Rao *et al*. (2017) [23] demonstrated that *B. licheniformis* CFR1 effectively detoxifies aflatoxin B_1_, achieving a remarkable 94.7% removal. Interestingly, the culture supernatant of this isolate exhibited significantly higher aflatoxin B_1_ degradation efficiency compared to cell lysate and intact cells, suggesting the involvement of secreted extracellular compounds. Similar to Chen *et al*. (2019) [34], who reported 70-80% inhibition of aflatoxin B_1_ production by synthetic peptides, the extracellular compounds from *B. siamensis* 3BS12-4 achieved a remarkable 96.11% removal in this study. While Suresh *et al*.(2020) [14] observed moderate aflatoxin degradation (60% and 34%) by cell-free extracts of *B. subtilis* and *Trametes versicolor*, attributed partly to oxidoreductases and hydrolases, the mechanism underlying the superior degradation ability of *B. siamensis* 3BS12-4 warrants further investigation.

### Biocontrol of Extracellular Compound on Peanut Seeds Under Storage Condition

**Fig. 4:**
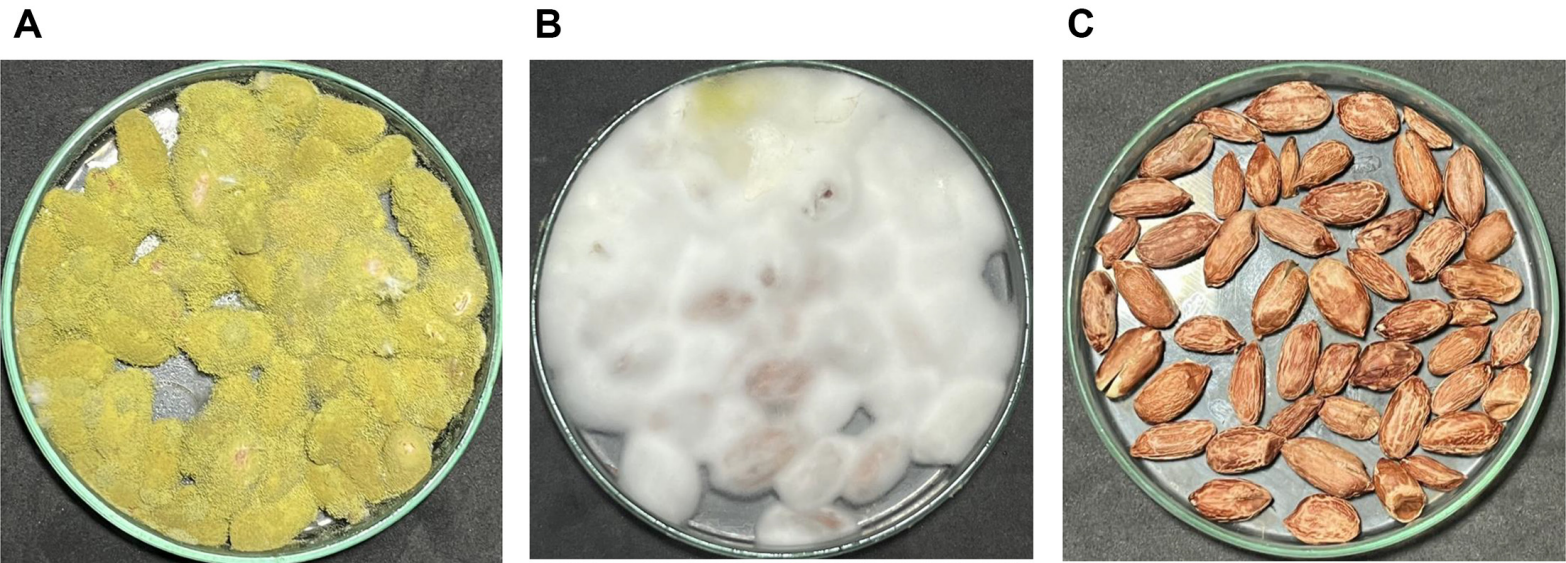
Effect of extracellular compounds from isolate 3BS12-4 on *A. flavus* growth on peanut seeds under storage conditions. Peanut seeds were treated with extracellular compounds from isolate 3BS12-4 followed by incubation at 30°C for 7 days. Panels: (**A**) non-treated control, (**B**) seeds treated with 0.512 g/ml of extracellular compounds, and (**C**) negative control not exposed to *A. flavus* or extracellular compounds.

Assessing the biocontrol potential of extracellular compounds from *B. siamensis* 3BS12-4 against *A. flavus* on peanut seeds, we observed a significant reduction in fungal growth after a 7-day incubation period. Treatment with 0.512 g/ml of the compounds resulted in a remarkable 97.93% inhibition of *A. flavus* colony count (1 × 10^6^ spore/g peanut seeds) compared to untreated controls. While complete inhibition was not achieved, these findings highlight the promising antifungal activity of *B. siamensis* 3BS12-4 compounds against *A. flavus* on peanut seeds (Fig. 4). Controlling the growth of *A. flavus* using antifungal compounds of *B. subtilis* B-FS06 on peanut seeds completely inhibited *A. flavus* growth at levels greater than 200 μg/g of antifungal compounds [35]. Shakeel *et al*. (2018) [45] reported that antifungal substances of *Streptomyces yanglinensis* 3-10 were evaluated inhibiting *A. flavus*, with no indication of *A. flavus* growth on peanut seeds. Reviewing several studies (*e.g.*, [31];[46]; [47]), it is evident that various *Bacillus* species hold significant promise for controlling *A. flavus* growth, thereby reducing mycotoxin contamination and enhancing agricultural product safety. The findings of the present study support this notion and demonstrate that the extracellular compounds produced by *B. siamensis* 3BS12-4 can significantly reduce *A. flavus* contamination on peanut seeds under storage conditions. While researchers worldwide have proposed various approaches for eliminating *A. flavus*, no single approach has proven universally effective due to the complex nature of this global threat. Combining multiple strategies tailored to specific contexts and challenges may offer the most effective solution [48].

*B. siamensis* is classified as Risk Group 1 by the German Collection of Microorganisms and Cell Cultures (DSMZ) [49]. This designation signifies a low or negligible risk to individual and public health, indicating a minimal likelihood of causing human or animal disease. Supporting this safety profile, Heo *et al*. (2021) [50] showed that *B. siamensis* B_2_8 is devoid of the genes for hemolysin, enterotoxin, and acquired antibiotic resistance. According to the genome, strain B_2_8 has genes for adhesion, survival in hostile environments, antibacterial activity against foodborne pathogens, production of vital amino acids, and improving human health. These findings highlight the potential of *B. siamensis* for various applications. The potential applications of *B. siamensis* in the feed, medical, and functional food industries warrant further investigation to explore its full potential as a safe and beneficial product.

In conclusion, this study establishes *B. siamensis* 3BS12-4 as a promising biological control agent for effectively inhibiting *A. flavus* colonization. This discovery paves the way for the development of environmentally friendly products to manage aflatoxin-producing fungi. While *B. siamensis* 3BS12-4 demonstrates significant inhibitory and reductive effects against *A. flavus* and aflatoxin, further research is necessary to fully characterize its antifungal potential. Future investigations will focus on the purification, identification, and characterization of the extracellular antifungal compounds, including their chemical features and biological properties. Additionally, elucidating the specific mechanisms underlying aflatoxin B_1_ degradation and determining the potential toxicity of any resulting byproducts are crucial next steps. Furthermore, exploring the possibility of *B. siamensis* 3BS12-4 producing lipopeptides through proteomic and LC-MS/MS analysis will provide valuable insights into the signaling pathways associated with its antifungal activity. Unveiling the specific antifungal compounds produced by *B. siamensis* 3BS12-4 remains a critical step towards unlocking its full potential for practical application.

## Supplemental Materials

Supplementary data for this paper are available on-line only at http://jmb.or.kr.



## Figures and Tables

**Table 1:** Inhibition of *A. flavus* spore germination by extracellular compounds from *B. siamensis* 3BS12-4.

	Extracellular compounds (2-fold dilutions (g/ml))										Value
	1.024	0.512	0.256	0.128	0.064	0.032	0.016	0.008	0.004	Control	
MIC test	-	-	+	+	+	+	+	+	+	+	0.512
MFC test	-	-	+	+	+	+	+	+	+	+	0.512

[+], growth; [-], no growth
