# Supplementary material for: Bacillus siamensis 3BS12-4 Extracellular Compounds as a Potential Biological Control Agent against Aspergillus flavus
Source: J Microbiol Biotechnol. 2024 Jun 30;34(8):1671–9. doi: 10.4014/jmb.2402.02053 (PMC11380522; doi:10.4014/jmb.2402.02053)
Supplement: Supplementary file 1 [file jmb-34-8-1671-supple.pdf]

J. Microbiol. Biotechnol. 2024. 34(8): 1671–1679
https://doi.org/10.4014/jmb.2402.02053

Supplementary Figure

A. Control treatment (aflatoxin B1 concentration with 32.93 ppb).

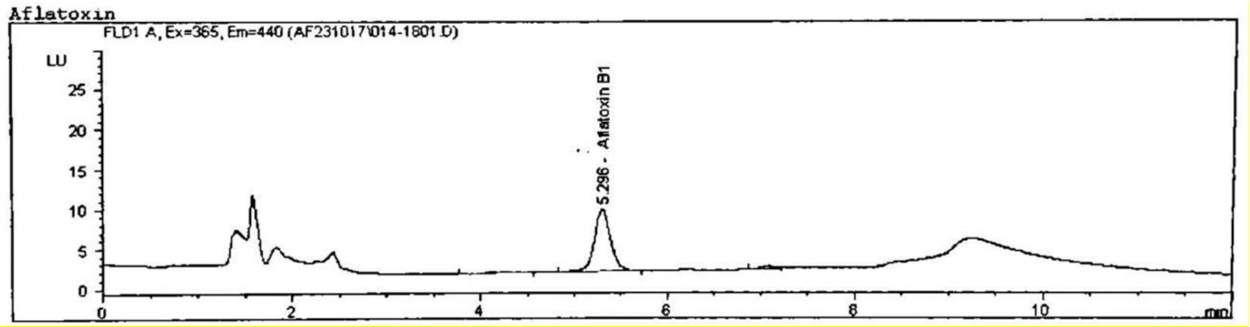

B. Aflatoxin B1 treated with extracellular compounds from *B. siamensis* 3BS12-4 (aflatoxin B1 concentration with 1.28 ppb).

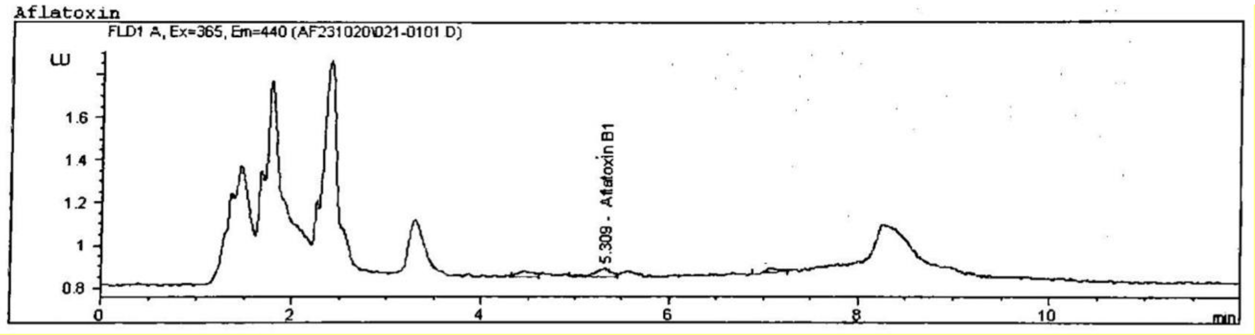

Fig. S1. HPLC chromatogram: A) aflatoxin B1 without extracellular compounds treatment (aflatoxin B1 concentration with 32.93 ppb) B) aflatoxin B1 treated with extracellular compounds from *B. siamensis* 3BS12-4 (aflatoxin B1 concentration with 1.28 ppb), after 3 days of incubation at 30°C.
